# Combining schizophrenia and depression polygenic risk scores improves the genetic prediction of lithium response in bipolar disorder patients

**DOI:** 10.1038/s41398-021-01702-2

**Published:** 2021-11-29

**Authors:** Klaus Oliver Schubert, Anbupalam Thalamuthu, Azmeraw T. Amare, Joseph Frank, Fabian Streit, Mazda Adl, Nirmala Akula, Kazufumi Akiyama, Raffaella Ardau, Bárbara Arias, Jean-Michel Aubry, Lena Backlund, Abesh Kumar Bhattacharjee, Frank Bellivier, Antonio Benabarre, Susanne Bengesser, Joanna M. Biernacka, Armin Birner, Cynthia Marie-Claire, Micah Cearns, Pablo Cervantes, Hsi-Chung Chen, Caterina Chillotti, Sven Cichon, Scott R. Clark, Cristiana Cruceanu, Piotr M. Czerski, Nina Dalkner, Alexandre Dayer, Franziska Degenhardt, Maria Del Zompo, J. Raymond DePaulo, Bruno Étain, Peter Falkai, Andreas J. Forstner, Louise Frisen, Mark A. Frye, Janice M. Fullerton, Sébastien Gard, Julie S. Garnham, Fernando S. Goes, Maria Grigoroiu-Serbanescu, Paul Grof, Ryota Hashimoto, Joanna Hauser, Urs Heilbronner, Stefan Herms, Per Hoffmann, Liping Hou, Yi-Hsiang Hsu, Stephane Jamain, Esther Jiménez, Jean-Pierre Kahn, Layla Kassem, Po-Hsiu Kuo, Tadafumi Kato, John Kelsoe, Sarah Kittel-Schneider, Ewa Ferensztajn-Rochowiak, Barbara König, Ichiro Kusumi, Gonzalo Laje, Mikael Landén, Catharina Lavebratt, Marion Leboyer, Susan G. Leckband, Mario Maj, Major Depressive Disorder Working Group of the Psychiatric Genomics Consortium, Mirko Manchia, Lina Martinsson, Michael J. McCarthy, Susan McElroy, Francesc Colom, Marina Mitjans, Francis M. Mondimore, Palmiero Monteleone, Caroline M. Nievergelt, Markus M. Nöthen, Tomas Novák, Claire O’Donovan, Norio Ozaki, Urban Ösby, Sergi Papiol, Andrea Pfennig, Claudia Pisanu, James B. Potash, Andreas Reif, Eva Reininghaus, Guy A. Rouleau, Janusz K. Rybakowski, Martin Schalling, Peter R. Schofield, Barbara W. Schweizer, Giovanni Severino, Tatyana Shekhtman, Paul D. Shilling, Katzutaka Shimoda, Christian Simhandl, Claire M. Slaney, Alessio Squassina, Thomas Stamm, Pavla Stopkova, Fasil Tekola-Ayele, Alfonso Tortorella, Gustavo Turecki, Julia Veeh, Eduard Vieta, Stephanie H. Witt, Gloria Roberts, Peter P. Zandi, Martin Alda, Michael Bauer, Francis J. McMahon, Philip B. Mitchell, Thomas G. Schulze, Marcella Rietschel, Bernhard T. Baune

**Affiliations:** 1 Discipline of Psychiatry, School of Medicine, University of Adelaide, Adelaide, SA Australia; 2 Northern Adelaide Local Health Network, Mental Health Services, Adelaide, SA Australia; 3 Centre for Healthy Brain Ageing (CHeBA), School of Psychiatry, University of New South Wales, Sydney, NSW Australia; 4 Department of Genetic Epidemiology in Psychiatry, Central Institute of Mental Health, Medical Faculty Mannheim, University of Heidelberg, Mannheim, Germany; 5 Department of Psychiatry and Psychotherapy, Charité—Universitätsmedizin Berlin, Campus Charité Mitte, Berlin, Germany; 6 Intramural Research Program, National Institute of Mental Health, National Institutes of Health, US Department of Health & Human Services, Bethesda, MD USA; 7 Department of Biological Psychiatry and Neuroscience, Dokkyo Medical University School of Medicine, Mibu, Tochigi Japan; 8 Unit of Clinical Pharmacology, Hospital University Agency of Cagliari, Cagliari, Italy; 9 Unitat de Zoologia i Antropologia Biològica (Dpt. Biologia Evolutiva, Ecologia i Ciències Ambientals), Facultat de Biologia and Institut de Biomedicina (IBUB), University of Barcelona, CIBERSAM, Barcelona, Spain; 10 Department of Psychiatry, Mood Disorders Unit, HUG—Geneva University Hospitals, Geneva, Switzerland; 11 Department of Molecular Medicine and Surgery, Karolinska Institute, Stockholm, Sweden, and Center for Molecular Medicine, Karolinska University Hospital, Stockholm, Sweden; 12 Department of Psychiatry, University of California San Diego, San Diego, CA USA; 13 INSERM UMR-S 1144, Université Paris Diderot, Département de Psychiatrie et de Médecine Addictologique, AP-HP, Groupe Hospitalier Saint-Louis-Lariboisière-F.Widal, Paris, France; 14 Bipolar Disorder Program, Institute of Neuroscience, Hospital Clinic, University of Barcelona, IDIBAPS, CIBERSAM, Barcelona, Catalonia Spain; 15 Department of Psychiatry and Psychotherapeutic Medicine, Research Unit for bipolar affective disorder, Medical University of Graz, Graz, Austria; 16 Department of Psychiatry and Psychology, Mayo Clinic, Rochester, MN USA; 17 The Neuromodulation Unit, McGill University Health Centre, Montreal, QC Canada; 18 Department of Psychiatry & Center of Sleep Disorders, National Taiwan University Hospital, Taipei, Taiwan; 19 Human Genomics Research Group, Department of Biomedicine, University Hospital Basel, Basel, Switzerland; 20 Institute of Human Genetics, University of Bonn and Department of Genomics, Life & Brain Center, Bonn, Germany; 21 Douglas Mental Health University Institute, McGill University, Montreal, QC Canada; 22 Psychiatric Genetic Unit, Poznan University of Medical Sciences, Poznan, Poland; 23 Department of Biomedical Sciences, University of Cagliari, Cagliari, Italy; 24 Department of Psychiatry and Behavioral Sciences, Johns Hopkins University, Baltimore, MD USA; 25 Department of Psychiatry and Psychotherapy, Ludwig-Maximilian-University Munich, Munich, Germany; 26 Department of Psychiatry (UPK), University of Basel, Basel, Switzerland; 27 Neuroscience Research Australia, Sydney, NSW Australia; 28 School of Medical Sciences, University of New South Wales, Sydney, NSW Australia; 29 Service de psychiatrie, Hôpital Charles Perrens, Bordeaux, France; 30 Department of Psychiatry, Dalhousie University, Halifax, NS Canada; 31 Biometric Psychiatric Genetics Research Unit, Alexandru Obregia Clinical Psychiatric Hospital, Bucharest, Romania; 32 Mood Disorders Center of Ottawa, Ottawa, ON Canada; 33 Molecular Research Center for Children’s Mental Development, United Graduate School of Child Development, Osaka University, Osaka, Japan; 34 Department of Psychiatry, Osaka University Graduate School of Medicine, Osaka, Japan; 35 Institute of Psychiatric Phenomics and Genomics (IPPG), University Hospital, LMU Munich, Munich, Germany; 36 HSL Institute for Aging Research, Harvard Medical School, Boston, MA USA; 37 Program for Quantitative Genomics, Harvard School of Public Health, Boston, MA USA; 38 Univ Paris Est Créteil, INSERM, IMRB, Translational Neuropsychiatry, Fondation FondaMental, Créteil, France; 39 Service de Psychiatrie et Psychologie Clinique, Centre Psychothérapique de Nancy—Université de Lorraine, Nancy, France; 40 Department of Public Health & Institute of Epidemiology and Preventive Medicine, College of Public Health, National Taiwan University, Taipei, Taiwan; 41 Department of Psychiatry & Behavioral Science, Juntendo University, Graduate School of Medicine, Tokyo, Japan; 42 Department of Psychiatry, Psychosomatic Medicine and Psychotherapy, University Hospital Frankfurt, Frankfurt, Germany; 43 Department of Adult Psychiatry, Poznan University of Medical Sciences, Poznan, Poland; 44 Department of Psychiatry and Psychotherapeutic Medicine, Landesklinikum Neunkirchen, Neunkirchen, Austria; 45 Department of Psychiatry, Hokkaido University Graduate School of Medicine, Sapporo, Japan; 46 Institute of Neuroscience and Physiology, the Sahlgrenska Academy at the Gothenburg University, Gothenburg, Sweden; 47 Department of Medical Epidemiology and Biostatistics, Karolinska Institutet, Stockholm, Sweden; 48 Inserm U955, Translational Neuro-Psychiatry laboratory, Université Paris Est Créteil (UPEC), AP-HP, Department of Psychiatry and Addictology of Mondor University Hospital, AP-HP, Fondation FondaMental, Créteil, France; 49 Office of Mental Health, VA San Diego Healthcare System, San Diego, CA USA; 50 Department of Psychiatry, University of Campania “Luigi Vanvitelli”, Naples, Italy; 51 Section of Psychiatry, Department of Medical Sciences and Public Health, University of Cagliari, Cagliari, Italy; 52 Department of Pharmacology, Dalhousie University, Halifax, NS Canada; 53 Department of Clinical Neurosciences, Karolinska Institutet, Stockholm, Sweden; 54 Department of Psychiatry, VA San Diego Healthcare System, San Diego, CA USA; 55 Department of Psychiatry, Lindner Center of Hope / University of Cincinnati, Mason, OH USA; 56 Mental Health Research Group, IMIM-Hospital del Mar, Barcelona, Catalonia Spain; 57 Centro de Investigación Biomédica en Red de Salud Mental (CIBERSAM), Instituto de Salud Carlos III, Madrid, Spain; 58 Departament de Genètica, Microbiologia i Estadística, Facultat de Biologia, Universitat de Barcelona, Barcelona, Spain; 59 Institut de Biomedicina de la Universitat de Barcelona (IBUB), Barcelona, Spain; 60 Centro de Investigación Biomédica en Salud Mental (CIBERSAM), Madrid, Spain; 61 Department of Medicine, Surgery and Dentistry “Scuola Medica Salernitana”, University of Salerno, Salerno, Italy; 62 National Institute of Mental Health, Klecany, Czech Republic; 63 Department of Psychiatry & Department of Child and Adolescent Psychiatry, Nagoya University Graduate School of Medicine, Nagoya, Japan; 64 Department of Neurobiology, Care Sciences, and Society, Karolinska Institutet and Center for Molecular Medicine, Karolinska University Hospital, Stockholm, Sweden; 65 Department of Psychiatry and Psychotherapy, University Hospital Carl Gustav Carus, Medical Faculty, Technische Universität Dresden, Dresden, Germany; 66 Montreal Neurological Institute and Hospital, McGill University, Montreal, QC Canada; 67 Department of Psychiatry, Dokkyo Medical University School of Medicine, Mibu, Tochigi Japan; 68 Bipolar Center Wiener Neustadt, Sigmund Freud University, Medical Faculty, Vienna, Austria; 69 Epidemiology Branch, Division of Intramural Population Health Research, Eunice Kennedy Shriver National Institute of Child Health and Human Development, National Institutes of Health, Bethesda, MD USA; 70 Department of Psychiatry, University of Perugia, Perugia, Italy; 71 School of Psychiatry, University of New South Wales, Sydney, Australia; 72 Department of Mental Health, Johns Hopkins Bloomberg School of Public Health, Baltimore, MD USA; 73 Department of Psychiatry and Psychotherapy, University Medical Center (UMG), Georg-August University Göttingen, Göttingen, Germany; 74 Department of Psychiatry and Psychotherapy, University of Münster, Münster, Germany; 75 Department of Psychiatry, Melbourne Medical School, University of Melbourne, Parkville, VIC Australia; 76 The Florey Institute of Neuroscience and Mental Health, The University of Melbourne, Parkville, VIC Australia

**Keywords:** Bipolar disorder, Pharmacogenomics

## Abstract

Lithium is the gold standard therapy for Bipolar Disorder (BD) but its effectiveness differs widely between individuals. The molecular mechanisms underlying treatment response heterogeneity are not well understood, and personalized treatment in BD remains elusive. Genetic analyses of the lithium treatment response phenotype may generate novel molecular insights into lithium’s therapeutic mechanisms and lead to testable hypotheses to improve BD management and outcomes. We used fixed effect meta-analysis techniques to develop meta-analytic polygenic risk scores (MET-PRS) from combinations of highly correlated psychiatric traits, namely schizophrenia (SCZ), major depression (MD) and bipolar disorder (BD). We compared the effects of cross-disorder MET-PRS and single genetic trait PRS on lithium response. For the PRS analyses, we included clinical data on lithium treatment response and genetic information for *n* = 2283 BD cases from the International Consortium on Lithium Genetics (ConLi^+^Gen; www.ConLiGen.org). Higher SCZ and MD PRSs were associated with poorer lithium treatment response whereas BD-PRS had no association with treatment outcome. The combined MET2-PRS comprising of SCZ and MD variants (MET2-PRS) and a model using SCZ and MD-PRS sequentially improved response prediction, compared to single-disorder PRS or to a combined score using all three traits (MET3-PRS). Patients in the highest decile for MET2-PRS loading had 2.5 times higher odds of being classified as poor responders than patients with the lowest decile MET2-PRS scores. An exploratory functional pathway analysis of top MET2-PRS variants was conducted. Findings may inform the development of future testing strategies for personalized lithium prescribing in BD.

## Introduction

Bipolar affective disorder (BD) is a severe and often chronic psychiatric illness, characterized by recurrent dysregulation of mood with alternating episodes of mania and depression. BD affects an estimated 48.8 million people globally and is associated with an early disease onset accounting for 9.9 million years of life lived with disability worldwide [1]. All-cause mortality and risk of suicide [2] are substantially increased in people with the disorder. Both genetic and environmental factors have been identified that contribute to the pathogenesis of BD [3] but the underlying molecular biology remains poorly understood, largely due to substantial genetic and clinical heterogeneity.

Lithium occupies a status as the ‘gold standard’ treatment amongst the mood-stabilizing medicines used for acute and maintenance therapy in BD [4–6]. It possesses strong anti-manic properties [7, 8] and is protective against further episodes of both manic and depressive polarity6, making it more effective in preventing re-hospitalizations than other BD medicines [9]. Furthermore, lithium has proven anti-suicidal properties [10]. Lithium is recommended as a first-line option for anti-manic and maintenance treatment by several clinical practice guidelines [11–14]. However, lithium therapy comes with several caveats. First, the therapeutic response to lithium in BD is highly heterogeneous. In acute mania, about 65% of patients respond at least partially to lithium monotherapy while 35% are refractory [15, 16]. In maintenance treatment, an excellent long-term response is reported only for ~30% of patients, whereas 30% have intermediate outcomes and 30% respond poorly [17]. Second, lithium is toxic at excessive doses and plasma levels need to be carefully monitored for the duration of treatment [18]. Third, lithium has long-term adverse effects including suppression of thyroid function and is associated with an elevated risk of renal failure [5].

To facilitate more personalized prescribing of lithium or alternative BD medicines, there is a need to define clinical and biological markers that could stratify BD patients into treatment response groups, ensuring that lithium is recommended at the earliest opportunity to patients most likely to respond, and is avoided in those who will likely derive no clinical benefits. Certain clinical factors (e.g. absence of physical co-morbidity [19, 20]; manic index episode [19]; family history of favourable lithium treatment [21] are associated with better treatment response, but none of these variables are sufficient to guide lithium prescribing on an individual basis. Among biological markers, both individual genetic variants (i.e. single nucleotide polymorphisms, SNPs [17] and the cumulative burden of many genetic variants (i.e. polygenic risk scores, PRS) have been identified that differentiate lithium response groups. We recently reported that BD patients with high genetic loading for schizophrenia, as calculated by PRS analysis, are less likely to have favourable lithium treatment outcomes than BD patients with low schizophrenia PRS [22]. Similarly, patients with high loadings for major depression (MDD) PRS were less likely to respond well to lithium than those with low MDD-PRS loading [23].

From these findings, several new research questions arise. First, does a combination of highly correlated genetic traits improve the predictive accuracy of the PRS approach? Previous studies have demonstrated that SCZ, BD, and MDD show substantial genetic overlap [24–26] and share biological pathways involved in disease pathophysiology [24, 25, 27]. The generation of multi-trait genomic risk scores from these genetically related disorders (SCZ, MDD, and BD) has been shown to improve diagnostic prediction accuracy [28], and therefore may be an avenue towards better translation of genetic findings into practice [29]. Second, while high SCZ and MDD-PRS were independently shown to predict poorer overall treatment response [22], other genomic signatures could predict better response, or different forms of response (e.g. level of agitation, depression, or cognition); hence, various combinations of PRS might help stratify or refine the response prediction. Third, related traits that are enriched with variants that predict the same direction of response (i.e. high load = poor response) might share certain key genetic variants, which influence response, enabling their molecular dissection.

In the current study, we investigated the contribution of variants from three genome-wide association studies (GWAS) in SCZ, MDD, and BD to lithium response in BD patients, and examined whether PRSs that are derived from cross-disorder meta-analysis of individual disorder GWAS improve genetic prediction. Further, we explored the relative individual contribution of disease-specific PRS to the combined PRS and investigated whether these were characterized by overlapping or divergent sets of genetic variants. Finally, to derive insights into molecular pathways that might influence lithium pharmacodynamics in BD, we conducted a pathway analysis of the top SNPs from the combined PRS analysis.

## Methods

### Discovery GWAS summary datasets

The GWAS summary statistics for SCZ, MDD, and BD were each obtained from the Psychiatric Genetics Consortium (PGC; http://www.med.unc.edu/pgc/). The SCZ dataset included 36,989 patients with SCZ and 113,075 healthy controls, including a subset of individuals with East Asian ancestry [26]. The MDD dataset was produced from a meta-analysis of seven cohorts (deCODE, Generation Scotland, GERA, iPSYCH, UK Biobank, PGC29 and 23andMe) containing 135,458 MD cases and 344,901 healthy controls [30]. The BD dataset was obtained from Stahl et al., 2019 [31], and the discovery sample was produced from a meta-analysis of 19,112 BD cases and 31,356 healthy controls after excluding 1242 individuals (1240 cases, 2 controls) who constituted the ConLi^+^Gen cohorts in the original data. Cross-disorder meta-analysis of the above summary statistics was performed using METACARPA [32], which accounts for unknown sample overlaps across the studies. If the summary statistics of multiple GWAS involve overlapping samples then the beta values across the GWAS are expected to be correlated (these correlations are functions of overlapping sample sizes) and failure to account these correlations would inflate the fixed effect inverse variance weighted met-analysis beta values [33]. When the exact number of samples overlapping across the studies are unknown, the correlations among betas can be empirically estimated. In a p-value based meta-analysis, the correlations between summary statistics were estimated using tetrachoric coefficients of truncated GWAS z-statistics instead of the Pearson correlations [34]. METACARPA uses this correlation matrix of beta values to account for the covariance between the GWAS beta values and implements computed meta-analysis statistics using Lin and Sullivan’s [33] method. The meta-summary statistics of MD, SCZ, and BD (MET3) and MD and SCZ (MET2) and Wald *p*-values produced METACARPA were employed for PRS calculation.

### Target study sample

For the PRS analysis, clinical data on lithium treatment response and genetic information were obtained from the International Consortium on Lithium Genetics (ConLi^+^Gen; www.ConLiGen.org). A sample of *n* = 2283 BD patients with complete phenotype information was included in the analysis [23].

### Target outcome

Lithium treatment response was assessed using the validated “Retrospective Criteria of Long-Term Treatment Response in Research Subjects with Bipolar Disorder” scale (the *Alda* scale) [35–37]. The instrument quantifies symptom improvement in BD by lithium over the course of treatment. Global improvement is rated with an “A” score (range 0–10), which is then weighted against five criteria (*Alda B* score) that assess the quality of evidence for the response score [17], to arrive at a total *Alda* score. For dichotomized assessment of treatment response, patients with a total score of 7 or higher were categorized as “good responders”, and the remainder were categorized as poor responders [17, 37]. For continuous assessment of treatment response, *total Alda* scores were used [38].

### Genotyping, quality control, and imputation

Genotyping and imputation in the ConLi^+^Gen cohorts have been described in detail elsewhere [17]. Briefly, genotyping across multiple cohorts was performed using several commercial SNP arrays (Affymetrix 6·0, Human610/660 W, HumanOmniExpress, HumanOmni1-Quad, HumanOmni2.5). The 1000 Genomes reference panel was used to impute additional genotypes as implemented in SHAPEIT2 [39] and minimac2 [40]. Quality control filters for the SNPs, including minor allele frequency (MAF) < 0.01, imputation quality score R-square < 0.6 and Hardy-Weinberg test of equilibrium for genotype frequencies (HWE) p-value ≤ 1e-6, were applied to the imputed data of each cohort. From the imputed dosage score, genotype calls for the filtered SNPs were derived and common sets of SNPs across the cohorts were merged using PLINK [41]. Merged genotype data from all the cohorts underwent additional post-merge quality control, and were used for creating PRSs.

### Polygenic Risk Score (PRS) analysis

The posterior effect sizes of GWAS and meta-analysis summary statistics were first obtained using the software PRS-CS [32]. PRS-CS uses the Bayesian regression framework to obtain the posterior effect sizes of the GWAS summary statistics with continuous shrinkage priors on the effect sizes and utilizing the linkage disequilibrium (LD) among the SNPs [42]. This approach results in a pruned set of posterior effect sizes for the summary statistics without the need to threshold the *p*-values for PRS calculation. For the current analysis, the precomputed LD pattern of the 1000 Genomes European reference panel and the default priors for the effect sizes were used. The PRS scores were generated using PLINK based on the posterior effect sizes instead of the actual effect sizes of the GWAS and meta-analysis.

### Statistical analysis

To assess the association of PRSs of individual traits (SCZ, BD, and MD), the cross-disorder meta-summary PRSs of SCZ, MD and BD (MET3), and SCZ and MD (MET2) with lithium treatment response, a binary logistic regression model was applied for the binary outcome (lithium response versus non-response). For association with *Alda total*, a tobit analysis model (censored regression) was used to account for the floor effect at zero. We used the following covariates for analysis: age, gender, 4 ethnicity principal components (PCs), site, and SNP chip type. The proportion of phenotypic variance explained by PRSs (partial R^2^) was calculated using the Nagelkerke method, as implemented in R package rsq [43]. R (version 4.0.0) [44] was used for data manipulations and statistical analyses. We also examined the joint effect of MDD-PRS and SCZ-PRS in a multivariate regression model with all the relevant covariates for comparison with MET2-PRS. The incremental R^2^ for the two PRS scores was computed as the difference in R^2^ of the models fit with and without the two PRS scores.

For all PRSs, we divided the study sample into deciles, ranging from the lowest polygenic load (1st decile, reference group) to the highest polygenic load (10th decile). Then, we compared lithium response rates in BD patients in the higher polygenic load deciles (2nd–10th deciles) with patients in the lowest polygenic load decile (1st decile).

We performed a power analysis for each PRS using the R package avengeme [45] with the input parameters heritability on liability, the number of independent SNPs, disease prevalence, and proportion of null markers. Heritability and prevalence for schizophrenia and bipolar disorder were obtained from Table 1 in Wray et al., 2010 [46]; the number of independent SNPs as produced by the PRS-CS package and the proportion of null markers were set as 90%.

**Table 1:** Characteristics of the patient sample.

Characteristic	Lithium responders (Alda Total Score ≥ 7)	Lithium non-responders (Alda Total Score ≤ 6)
*N* (%)	637 (27.9)	1646 (72.1)
Female *N* (%)	352 (55.3)	950 (57.7)
Age mean (SD)	50.23 (14.71)	45.92 (13.47)
Alda A mean (SD)	9.23 (0.79)	5.07 (2.7)
Alda B mean (SD)	1.14 (0.94)	3.04 (1.61)

*SD* standard deviation.

### Functional analyses

To explore the biological context and potential mechanistic underpinnings of the SNPs discovered from the cross-disorder meta-analyses, we performed pathway analyses using Qiagen’s Ingenuity Pathway Analysis (IPA®, QIAGEN Redwood City, CA, USA, www.qiagen.com/ingenuity). To prepare the input genes for IPA®, we followed a two-step approach: first, all SNPs from the MET2-PRS that showed associations with both SCZ and MDD at meta-analysis threshold *p* < 5 × 10^−8^ were selected; second, SNPs from step 1 were mapped to their hosting genes using ANNOVAR software [47]. For intergenic SNPs, ANNOVAR provides distance to the nearest upstream and downstream gene. The SNPs were mapped to the closest genes using this distance (range: 0-796688 BP). This final list of mapped genes was entered into IPA® (content version: 52912811, 2020).

IPA compares the proportion of input genes mapping to a biological pathway to the reference gene list from the ingenuity databases. Molecule relationships previously experimentally observed in human, mouse, rat, and uncategorized species were included. The significance of the over-represented canonical pathways and functional networks is determined using the right-tailed Fisher’s exact test and later adjusted for multiple testing using the Benjamini-Hochberg (BH) method. Significant results are determined at BH adjusted *p*-value < 0.01.

## Results

### Sample

Details of the ConLi^+^Gen sample of patients with BD have been published previously [17]. For the current analyses, genetic and clinical data from *n* = 2283 patients were used (57% females). 27.9% of patients were classified as excellent lithium responders (Alda Total Score ≥ 7), whereas 72.1% were classified as non-responders (Alda Total Score ≤ 6) (Table 1).

### Associations of individual and combined SCZ, MDD, and BD-PRS with lithium treatment response

**Fig. 1:**
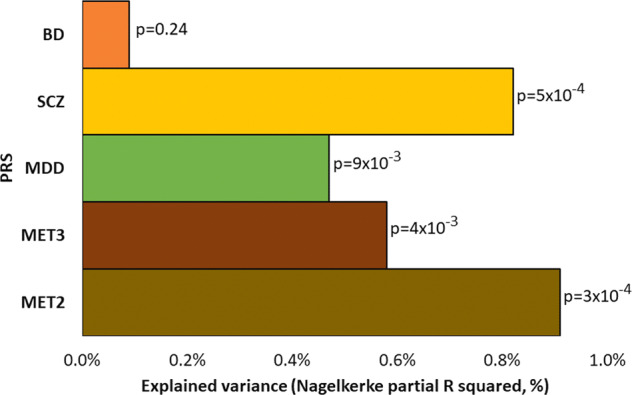
The associations of polygenic risk scores (PRS) for bipolar disorder (BD), schizophrenia (SCZ), major depression (MDD), meta-SCZ/MDD/BD (MET3), and meta-SCZ/MDD (MET2) with dichotomous lithium treatment response (Alda total ≥ 7). The *x*-axis refers to the percentage of explained variance in treatment response to lithium accounted for by the PRS (Nagelkerke partial R^2^). The *y*-axis plots the PRS for BD, SCZ, MDD, MET3, and MET2. Each bar is labelled with the *p*-values for the association between the PRS and lithium treatment response.

The dichotomous lithium treatment response outcome (*Alda total* ≥ 7, indicating excellent vs non-responders) was significantly associated with SCZ-PRS (*p* = 0.0005) and MDD-PRS (*p* = 0.009), but not with BD-PRS (*p* = 0.24) (Fig. 1, Supplementary Table 1). Power analyses for each PRS indicated that the sample sizes required for the training dataset for 80% power were similar for schizophrenia (*n* = 5537) and bipolar disorder (*n* = 5741), suggesting that the observed lack of association between BD-PRS and lithium response may not be due to lack of power.

Significant associations were also shown for MET3-PRS (comprised of SCZ, MDD, and BD; *p* = 0.004) and MET2-PRS (comprised of SCZ and MDD; *p* = 0.0003). Each of these associations remained significant after adjustment for multiple testing (*p*-threshold = 0.05/5 = 0.01). The proportion of phenotype variance explained by MET2-PRS (partial R^2^ = 0.91%) was higher than for SCZ-PRS (partial R^2^ = 0.82%), MDD-PRS (partial R^2^ = 0.47%), and MET3-PRS (partial R^2^ = 0.58%) (Fig. 1, Supplementary Table 1).

We also examined the joint effect of MDD-PRS and SCZ-PRS in a multivariate regression model with all the relevant covariates for comparison with MET2-PRS. The incremental R^2^ for the two PRS scores was found to be 0.92%, similar to MET2-PRS R^2^. The joint *p*-value for the two scores was *p* = 0.0002, again similar to the MET2-PRS *p*-value (supplementary Table 1).

The continuous lithium response outcome *Alda total*, using a tobit regression model, was significantly associated with SCZ-PRS (*p* = 0.0095), MD-PRS (*p* = 0.0029), MET3-PRS (0.0044), and MET2-PRS (*p* = 0.0003). BD-PRS was not associated with *Alda total* (Supplementary Table 2). PRS beta values for all associations were negative, indicating a consistent direction of effect of PRS for SCZ, MDD, MET3, and MET2 on lithium response as measured by *Alda total*.

To further evaluate the impact of the individual and combined PRS on lithium treatment response, we divided the study population into deciles based on their polygenic loading. Samples were grouped based on the deciles for PRS for BD, SCZ, MDD, MET3, and MET2 (*n* = 228 in each decile group). Decile group 1 (reference group) contained individuals with the lowest genetic loading for the respective PRS, whereas decile group 10 represented those with the highest loading.

**Fig. 2:**
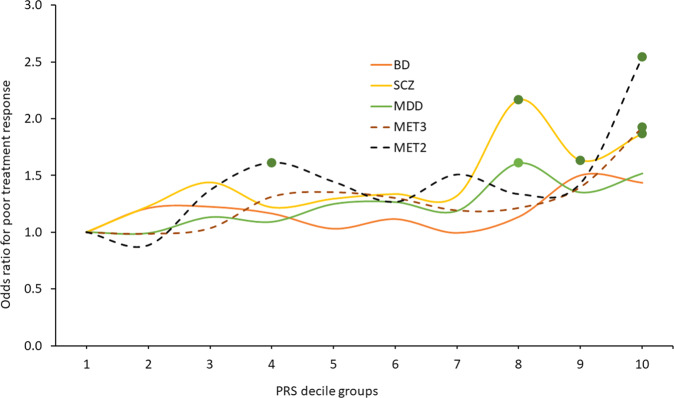
Odds ratios (ORs) for unfavourable treatment response to lithium (Alda score ≤ 6) in patients with BD. ORs are derived by comparing patients with higher polygenic loads (PRS deciles 2–10) for BD (orange line), SCZ (yellow line), MDD (green line), meta-(SCZ/MDD/BD) (MET3, maroon dashed line), and meta-(SCZ/MDD) (MET2, brown dotted line) with patients with the lowest respective polygenic loads (PRS 1st decile). Dots indicate *p* < 0.05. Analyses were adjusted for gender, age, 4 PCs, site, and SNP chip type.

Individuals with higher polygenic loading for any of the tested PRS except BD-PRS were at increased risk of experiencing a poor therapeutic response to lithium (Fig. 2). Compared to the low PRS (1st decile) reference groups, odds ratios (ORs) for poor response were significantly different (*p* < 0.05) for SCZ-PRS (8th decile: OR = 2.16, *p* = 0.001; 9th decile: OR 1.63, *p* = 0.04; 10th decile: OR = 1.87, *p* = 0.04), MDD-PRS (8th decile: OR = 1.61, *p* = 0.04), MET3-PRS (10th decile: OR = 1.92, *p* = 0.03), and MET2-PRS (4th decile: OR = 1.61, *p* = 0.03; 10th decile: OR = 2.54, *p* = 0.002). (Fig. 2, Supplementary Table 3).

Pearson correlations between disease-specific and meta-PRS were calculated. As shown in Supplementary Table 4, the correlation between SCZ-PRS and MET2-PRS was r = 0.83, whereas MDD-PRS and MET2-PRS had a correlation of r = 0.6.

### Exploratory functional analysis of top SCZ-MDD-PRS (MET2) variants

Eight thousands six hundred nineteen SNPs from the MDD and SCZ meta-analysis in METACARPA (MET2) at p ≤ 5 × 10^−8^ and in consistent direction in both GWAS studies were selected to explore the biological context of overlapping risk alleles (Supplementary Table 5). Of note, a PRS calculated from these selected 8619 SNPs was not by itself associated with lithium response (data not shown). By mapping SNPs to hosting- and nearby genes (distance to nearest gene is shown in Supplementary Table 5), we derived a list of 270 genes (Supplementary Table 6). 106 (41%) of these genes intersected with SCZ genes (GWAS *p*-value ≤ 5 × 10^−8^); 73 (29%) intersected with MDD genes (GWAS *p*-value ≤ 5 × 10^−8^); 37 (14%) intersected with both SCZ and MDD genes (Supplementary Table 6). Of the 270 MET2 genes, IPA® could unequivocally identify 256 genes in its database for enrichment and pathway analysis (Supplementary Table 7).

IPA® examined six categories of enrichment in which genes from the MET2 list were over-represented, including: *top canonical pathways; upstream regulators; diseases & disorders; molecular & cellular functions; physiological system functions; and networks* (Supplementary Table 8). The majority of these associations were driven by genes involved in histone biology (e.g. H4C1, H4C2, H4C3, H4C4, H4C8, H4C11, H4C12, H4C13; H3C2, H3C3, H3C8, H3C10, H3C11, H3C12; H1-1, H1-2, H1-4, H1-5, H1-6). Over-representation of MET2 genes was found for IPA® *disease-related pathways* in endocrine system disorders, gastrointestinal disease, immunological disease, metabolic disease, and organismal injury and abnormalities (Supplementary Table 8). Associations with the first three disease categories (endocrine, gastrointestinal, and immunological) was most strongly driven by MET2 genes over-represented in IPA®-defined genesets relating to insulin-dependent diabetes mellitus, diabetes mellitus, and glucose metabolism disorder (Supplementary Table 9).

**Fig. 3:**
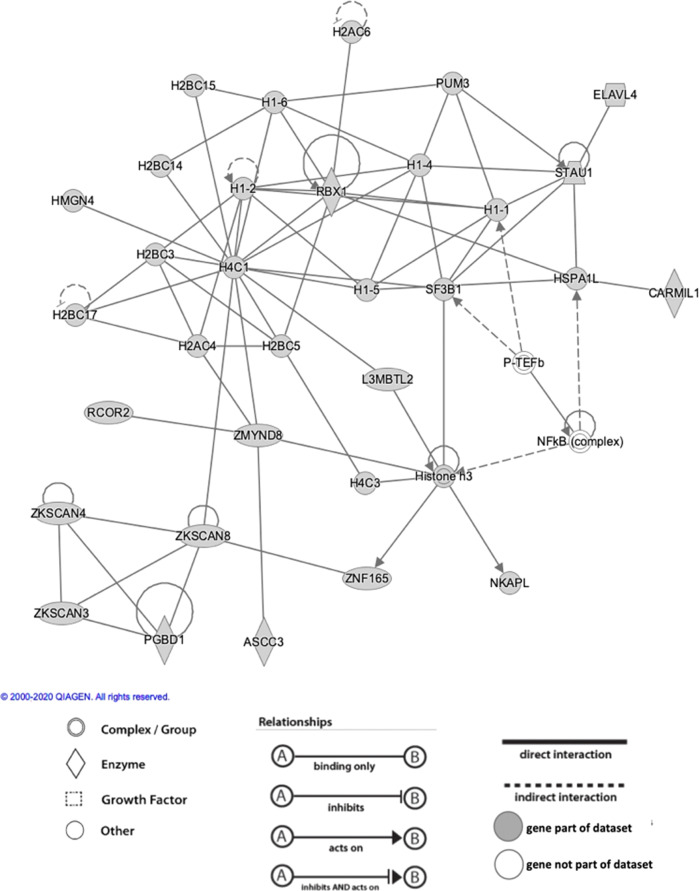
IPA® top network of MET2 genes associated with SCZ and MDD at *p* < 5 × 10^−8^. Annotated network functions include endocrine system disorders, gastrointestinal disease, immunological disease. H4 clustered histone 1 (H4C1), histone H3, and E3 ubiquitin-protein ligase (RBX1) are identified as nodes with most network interactions.

IPA® also calculated *networks* of the functionally most connected MET2 genes. Network 1 (IPA® score 67) was annotated to *endocrine system disorders, gastrointestinal disease, and immunological disease*, and identified H4 clustered histone 1 (H4C1), histone h3, and E3 ubiquitin-protein ligase (RBX1) as nodes with the highest number of network connections (Fig. 3, Supplementary Table 8). Network 2 (IPA® score 39) was annotated with *cell cycle, cellular assembly and organization, and DNA replication, recombination, and repair*. Adenosine triphosphate (ATP), estrogen receptor, and EP300 (encoding p300, a histone acetyltransferase) were identified as nodes with most network interactions (Supplementary Fig. 1). Networks 3, 4, and 5 (each with IPA® score 32), respectively, identified tumor necrosis factor (TNF), extracellular-signal-regulated kinases (ERK1/2), and heat shock protein family D member 1 (HSPD1) as most highly connected nodes (Supplementary Figs. 2–4).

## Discussion

To our knowledge, this is the first study analysing both individual *and* shared contributions of polygenic risk scores (PRS) for SCZ, MDD, and BD to the therapeutic response to lithium in individuals with bipolar disorder. Placing continuous shrinkage (CS) priors on SNP effect sizes for calculating PRS [42], we found that high genetic loadings for SCZ and MDD but not BD reduce patients’ likelihood of optimal clinical lithium response. We also found that a *combined* PRS derived from GWAS meta-analysis of MDD and SCZ (MET2-PRS) improves genetic response prediction, compared with individual disorder PRS and with a combined PRS comprising SCZ, MDD, and BD (MET3). The 228 patients with the highest MET2 loading in our cohort (top 10%) had 2.5 times greater odds of experiencing a poor clinical response to lithium, compared to the 228 patients with the lowest loading (bottom 10%). Exploratory bioinformatic pathway analysis (IPA®) of the genes containing MET2 variants most strongly associated with both MDD and SCZ (*p* < 5 × 10^−8^) implicated histone biology and genesets relating to metabolic disorders such as diabetes mellitus.

Our finding of negative associations between high loadings of SCZ-PRS and MDD-PRS with poor lithium response confirms our previous studies investigating these psychiatric traits in the ConLi^+^Gen cohort [22, 23]. Results are consistent with the clinical observation of poorer lithium response in BD patients who have a family history of SCZ, as opposed to a family history of BD [36], and support the idea that better lithium responsiveness is associated with a ‘core’ bipolar phenotype in the form of manic depression [5]. The specificity of SCZ- and MDD-PRS but not BD-PRS in predicting response is noteworthy, especially on the background of an 80% overlap in our study between SCZ-PRS and BD-PRS contrasting a 13% overlap between SCZ-PRS and MDD-PRS. Findings suggest that the genetic variants which relate to lithium responsiveness are operating independently to the risk of BD. How such trait-specificity is explained needs to be explored in future research. For example, there could be shared and separate sets of SCZ and MDD-risk alleles influencing lithium treatment response that are not contained within the BD-PRS. Alternatively, certain variants within the BD-PRS could be protective against the negative impact of shared SCZ and MDD-risk alleles.

The superior performance of MET2 in predicting lithium response, as compared to individual disorders, is consistent with previous work showing that multi-trait genomic risk scores can improve prediction accuracy [28]. It is notable that MET3, showing a 96% overlap with MET2, was not superior to PRS derived for individual diseases. Therefore, for genetic prediction of clinical outcomes, our study encourages a workflow that assesses single-trait associations first, and in a second step considers combined PRS using significantly associated traits only, irrespective of a priori single PRS correlations.

Our exploratory IPA® pathway analysis of top variants associated with both SCZ and MDD within the MET2-PRS proposes several overlapping biological mechanisms in these disorders. Some of these shared SCZ-MDD pathways may, in turn, play a role in lithium response. For example, the high prevalence of histone genes within our SCZ-MDD gene set may support findings from in vitro, animal and human studies demonstrating an influence of lithium on gene expression through epigenetic mechanisms including histone modification (for review, see ref. [48]) and the recent notion of epigenetic signatures as a potential molecular predictor of lithium response in BD [49]. Studies have shown that lithium treatment increases acetylation and methylation of histone H3 in rat hippocampus [50], and that it suppresses histone deacetylases (HDACs) [51]. Similarly, the high prevalence of genes within MET2 linked to diabetes mellitus and glucose metabolism echoes clinical-epidemiological studies showing that BD patients with comorbid diabetes mellitus or insulin resistance have poorer responses to lithium [20].

Our study has a number of limitations. First, the variance in lithium response that can be explained by the PRS method remains small (partial R^2^ = 0.91%), and it is unlikely that PRSs as stand-alone biological markers can be translated into clinical practice. However, genetic scores might be used in concert with other biological parameters and with clinical patient characteristics to form predictive algorithms of lithium treatment response. Machine-learning approaches to generate such multivariate algorithms have recently shown promise [52]. Second, our study used a lithium response measure (the Alda scale) focusing on long-term protection from manic and depressive relapses. Other forms of response to lithium (e.g. reduction of agitation in acute mania, reduction in overall suicidality) are less well captured by the Alda scale but are clinically important. Third, SZ-PRS and MDD-PRS are more statistically powerful than BD-PRS as they have been calculated on larger populations. This might partly explain the low contribution of BD-PRS in discriminating responders and non-responders, even though our power analysis indicated that the patient numbers required for each PRS are similar and were sufficient in our study. Forth, our functional analysis of top MET2 variants is only exploratory, as a direct association of these variants to lithium response (in addition to their association with SCZ and MDD) cannot be demonstrated through the methods employed in this study. Further, we chose a heuristic *p*-value cut-off for inclusion of MET2 variants into our bioinformatic pathways analysis, which employed the traditional threshold for genome-wide significance (*p* < 5 × 10^−8^). More generous cut-offs, resulting in larger gene lists for input into IPA® analysis may have identified different or additional functional links to MET2 genes.

## Conclusions

The application of multi-trait genomic risk scores revealed that a meta-PRS generated from a meta-GWAS of SCZ- and MDD-risk alleles (MET2-PRS), but not including BD-risk alleles, improves genetic prediction accuracy of the lithium response phenotype in BD compared to single-disorder PRS. High MET2-PRS loading is associated with poorer lithium response. Multi-trait PRS may inform the development of future biological testing strategies for personalized lithium prescribing in BD and may assist in the search for central therapeutic molecular mechanisms of this important drug.

## Supplementary information

Supplemental material

Supplementary tables

Supplementary information
