# Correction: Combining schizophrenia and depression polygenic risk scores improves the genetic prediction of lithium response in bipolar disorder patients

**DOI:** 10.1038/s41398-022-02037-2

**Published:** 2022-07-11

**Authors:** Klaus Oliver Schubert, Anbupalam Thalamuthu, Azmeraw T. Amare, Joseph Frank, Fabian Streit, Mazda Adl, Nirmala Akula, Kazufumi Akiyama, Raffaella Ardau, Bárbara Arias, Jean-Michel Aubry, Lena Backlund, Abesh Kumar Bhattacharjee, Frank Bellivier, Antonio Benabarre, Susanne Bengesser, Joanna M. Biernacka, Armin Birner, Cynthia Marie-Claire, Micah Cearns, Pablo Cervantes, Hsi-Chung Chen, Caterina Chillotti, Sven Cichon, Scott R. Clark, Cristiana Cruceanu, Piotr M. Czerski, Nina Dalkner, Alexandre Dayer, Franziska Degenhardt, Maria Del Zompo, J. Raymond DePaulo, Bruno Étain, Peter Falkai, Andreas J. Forstner, Louise Frisen, Mark A. Frye, Janice M. Fullerton, Sébastien Gard, Julie S. Garnham, Fernando S. Goes, Maria Grigoroiu-Serbanescu, Paul Grof, Ryota Hashimoto, Joanna Hauser, Urs Heilbronner, Stefan Herms, Per Hoffmann, Liping Hou, Yi-Hsiang Hsu, Stephane Jamain, Esther Jiménez, Jean-Pierre Kahn, Layla Kassem, Po-Hsiu Kuo, Tadafumi Kato, John Kelsoe, Sarah Kittel-Schneider, Ewa Ferensztajn-Rochowiak, Barbara König, Ichiro Kusumi, Gonzalo Laje, Mikael Landén, Catharina Lavebratt, Marion Leboyer, Susan G. Leckband, Mario Maj, Major Depressive Disorder Working Group of the Psychiatric Genomics Consortium, Mirko Manchia, Lina Martinsson, Michael J. McCarthy, Susan McElroy, Francesc Colom, Marina Mitjans, Francis M. Mondimore, Palmiero Monteleone, Caroline M. Nievergelt, Markus M. Nöthen, Tomas Novák, Claire O’Donovan, Norio Ozaki, Urban Ösby, Sergi Papiol, Andrea Pfennig, Claudia Pisanu, James B. Potash, Andreas Reif, Eva Reininghaus, Guy A. Rouleau, Janusz K. Rybakowski, Martin Schalling, Peter R. Schofield, Barbara W. Schweizer, Giovanni Severino, Tatyana Shekhtman, Paul D. Shilling, Katzutaka Shimoda, Christian Simhandl, Claire M. Slaney, Alessio Squassina, Thomas Stamm, Pavla Stopkova, Fasil Tekola-Ayele, Alfonso Tortorella, Gustavo Turecki, Julia Veeh, Eduard Vieta, Stephanie H. Witt, Gloria Roberts, Peter P. Zandi, Martin Alda, Michael Bauer, Francis J. McMahon, Philip B. Mitchell, Thomas G. Schulze, Marcella Rietschel, Bernhard T. Baune

**Affiliations:** 1 Discipline of Psychiatry, School of Medicine, University of Adelaide, Adelaide, SA Australia; 2 Northern Adelaide Local Health Network, Mental Health Services, Adelaide, SA Australia; 3 Centre for Healthy Brain Ageing (CHeBA), School of Psychiatry, University of New South Wales, Sydney, NSW Australia; 4 Department of Genetic Epidemiology in Psychiatry, Central Institute of Mental Health, Medical Faculty Mannheim, University of Heidelberg, Mannheim, Germany; 5 Department of Psychiatry and Psychotherapy, Charité—Universitätsmedizin Berlin, Campus Charité Mitte, Berlin, Germany; 6 Intramural Research Program, National Institute of Mental Health, National Institutes of Health, US Department of Health & Human Services, Bethesda, MD USA; 7 Department of Biological Psychiatry and Neuroscience, Dokkyo Medical University School of Medicine, Mibu, Tochigi Japan; 8 Unit of Clinical Pharmacology, Hospital University Agency of Cagliari, Cagliari, Italy; 9 Unitat de Zoologia i Antropologia Biològica (Dpt. Biologia Evolutiva, Ecologia i Ciències Ambientals), Facultat de Biologia and Institut de Biomedicina (IBUB), University of Barcelona, CIBERSAM, Barcelona, Spain; 10 Department of Psychiatry, Mood Disorders Unit, HUG—Geneva University Hospitals, Geneva, Switzerland; 11 Department of Molecular Medicine and Surgery, Karolinska Institute, Stockholm, Sweden, and Center for Molecular Medicine, Karolinska University Hospital, Stockholm, Sweden; 12 Department of Psychiatry, University of California San Diego, San Diego, CA USA; 13 INSERM UMR-S 1144, Université Paris Diderot, Département de Psychiatrie et de Médecine Addictologique, AP-HP, Groupe Hospitalier Saint-Louis-Lariboisière-F.Widal, Paris, France; 14 Bipolar Disorder Program, Institute of Neuroscience, Hospital Clinic, University of Barcelona, IDIBAPS, CIBERSAM, Barcelona, Catalonia Spain; 15 Department of Psychiatry and Psychotherapeutic Medicine, Research Unit for bipolar affective disorder, Medical University of Graz, Graz, Austria; 16 Department of Psychiatry and Psychology, Mayo Clinic, Rochester, MN USA; 17 The Neuromodulation Unit, McGill University Health Centre, Montreal, QC Canada; 18 Department of Psychiatry & Center of Sleep Disorders, National Taiwan University Hospital, Taipei, Taiwan; 19 Human Genomics Research Group, Department of Biomedicine, University Hospital Basel, Basel, Switzerland; 20 Institute of Human Genetics, University of Bonn and Department of Genomics, Life & Brain Center, Bonn, Germany; 21 Douglas Mental Health University Institute, McGill University, Montreal, QC Canada; 22 Psychiatric Genetic Unit, Poznan University of Medical Sciences, Poznan, Poland; 23 Department of Biomedical Sciences, University of Cagliari, Cagliari, Italy; 24 Department of Psychiatry and Behavioral Sciences, Johns Hopkins University, Baltimore, MD USA; 25 Department of Psychiatry and Psychotherapy, Ludwig-Maximilian-University Munich, Munich, Germany; 26 Department of Psychiatry (UPK), University of Basel, Basel, Switzerland; 27 Neuroscience Research Australia, Sydney, NSW Australia; 28 School of Medical Sciences, University of New South Wales, Sydney, NSW Australia; 29 Service de psychiatrie, Hôpital Charles Perrens, Bordeaux, France; 30 Department of Psychiatry, Dalhousie University, Halifax, NS Canada; 31 Biometric Psychiatric Genetics Research Unit, Alexandru Obregia Clinical Psychiatric Hospital, Bucharest, Romania; 32 Mood Disorders Center of Ottawa, Ottawa, ON Canada; 33 Molecular Research Center for Children’s Mental Development, United Graduate School of Child Development, Osaka University, Osaka, Japan; 34 Department of Psychiatry, Osaka University Graduate School of Medicine, Osaka, Japan; 35 Institute of Psychiatric Phenomics and Genomics (IPPG), University Hospital, LMU Munich, Munich, Germany; 36 HSL Institute for Aging Research, Harvard Medical School, Boston, MA USA; 37 Program for Quantitative Genomics, Harvard School of Public Health, Boston, MA USA; 38 Univ Paris Est Créteil, INSERM, IMRB, Translational Neuropsychiatry, Fondation FondaMental, Créteil, France; 39 Service de Psychiatrie et Psychologie Clinique, Centre Psychothérapique de Nancy—Université de Lorraine, Nancy, France; 40 Department of Public Health & Institute of Epidemiology and Preventive Medicine, College of Public Health, National Taiwan University, Taipei, Taiwan; 41 Department of Psychiatry & Behavioral Science, Juntendo University, Graduate School of Medicine, Tokyo, Japan; 42 Department of Psychiatry, Psychosomatic Medicine and Psychotherapy, University Hospital Frankfurt, Frankfurt, Germany; 43 Department of Adult Psychiatry, Poznan University of Medical Sciences, Poznan, Poland; 44 Department of Psychiatry and Psychotherapeutic Medicine, Landesklinikum Neunkirchen, Neunkirchen, Austria; 45 Department of Psychiatry, Hokkaido University Graduate School of Medicine, Sapporo, Japan; 46 Institute of Neuroscience and Physiology, the Sahlgrenska Academy at the Gothenburg University, Gothenburg, Sweden; 47 Department of Medical Epidemiology and Biostatistics, Karolinska Institutet, Stockholm, Sweden; 48 Inserm U955, Translational Neuro-Psychiatry laboratory, Université Paris Est Créteil (UPEC), AP-HP, Department of Psychiatry and Addictology of Mondor University Hospital, AP-HP, Fondation FondaMental, Créteil, France; 49 Office of Mental Health, VA San Diego Healthcare System, San Diego, CA USA; 50 Department of Psychiatry, University of Campania “Luigi Vanvitelli”, Naples, Italy; 51 Section of Psychiatry, Department of Medical Sciences and Public Health, University of Cagliari, Cagliari, Italy; 52 Department of Pharmacology, Dalhousie University, Halifax, NS Canada; 53 Department of Clinical Neurosciences, Karolinska Institutet, Stockholm, Sweden; 54 Department of Psychiatry, VA San Diego Healthcare System, San Diego, CA USA; 55 Department of Psychiatry, Lindner Center of Hope / University of Cincinnati, Mason, OH USA; 56 Mental Health Research Group, IMIM-Hospital del Mar, Barcelona, Catalonia Spain; 57 Centro de Investigación Biomédica en Red de Salud Mental (CIBERSAM), Instituto de Salud Carlos III, Madrid, Spain; 58 Departament de Genètica, Microbiologia i Estadística, Facultat de Biologia, Universitat de Barcelona, Barcelona, Spain; 59 Institut de Biomedicina de la Universitat de Barcelona (IBUB), Barcelona, Spain; 60 Centro de Investigación Biomédica en Salud Mental (CIBERSAM), Madrid, Spain; 61 Department of Medicine, Surgery and Dentistry “Scuola Medica Salernitana”, University of Salerno, Salerno, Italy; 62 National Institute of Mental Health, Klecany, Czech Republic; 63 Department of Psychiatry & Department of Child and Adolescent Psychiatry, Nagoya University Graduate School of Medicine, Nagoya, Japan; 64 Department of Neurobiology, Care Sciences, and Society, Karolinska Institutet and Center for Molecular Medicine, Karolinska University Hospital, Stockholm, Sweden; 65 Department of Psychiatry and Psychotherapy, University Hospital Carl Gustav Carus, Medical Faculty, Technische Universität Dresden, Dresden, Germany; 66 Montreal Neurological Institute and Hospital, McGill University, Montreal, QC Canada; 67 Department of Psychiatry, Dokkyo Medical University School of Medicine, Mibu, Tochigi Japan; 68 Bipolar Center Wiener Neustadt, Sigmund Freud University, Medical Faculty, Vienna, Austria; 69 Epidemiology Branch, Division of Intramural Population Health Research, Eunice Kennedy Shriver National Institute of Child Health and Human Development, National Institutes of Health, Bethesda, MD USA; 70 Department of Psychiatry, University of Perugia, Perugia, Italy; 71 School of Psychiatry, University of New South Wales, Sydney, Australia; 72 Department of Mental Health, Johns Hopkins Bloomberg School of Public Health, Baltimore, MD USA; 73 Department of Psychiatry and Psychotherapy, University Medical Center (UMG), Georg-August University Göttingen, Göttingen, Germany; 74 Department of Psychiatry and Psychotherapy, University of Münster, Münster, Germany; 75 Department of Psychiatry, Melbourne Medical School, University of Melbourne, Parkville, VIC Australia; 76 The Florey Institute of Neuroscience and Mental Health, The University of Melbourne, Parkville, VIC Australia

**Keywords:** Bipolar disorder, Pharmacogenomics

Correction to: *Translational Psychiatry* 10.1038/s41398-021-01702-2, published online 29 November 2021

The following Funding information was missing from this article.

The study was supported by the German Federal Ministry of Education and Research (BMBF) through ERA-NET NEURON grants “SynSchiz—Linking synaptic dysfunction to disease mechanisms in schizophrenia—a multilevel investigation” (01EW1810 to MR) and “Impact of Early life MetaBolic and psychosocial strEss on susceptibility to mental Disorders; from converging epigenetic signatures to novel targets for therapeutic intervention” (01EW1904 to MR); and by the German Research Foundation (DFG grants FOR2107; RI908/11-2 to MR; WI 3439/3-2 to SHW). ATA is supported by 2019–2021 National Alliance for Research on Schizophrenia and Depression (NARSAD) Young Investigator Grant from the Brain & Behaviour Research Foundation (BBRF) and National Health and Medical Research Council (NHMRC) Emerging Leadership Investigator Grant 2021–2008000.

The original article has been corrected.

