# Supplementary material for: Combining schizophrenia and depression polygenic risk scores improves the genetic prediction of lithium response in bipolar disorder patients
Source: Transl Psychiatry. 2021 Nov 29;11:606. doi: 10.1038/s41398-021-01702-2 (PMC8630000; doi:10.1038/s41398-021-01702-2)
Supplement: Supplementary file 1 — Supplemental material [file 41398_2021_1702_MOESM1_ESM.docx]

**Combining schizophrenia and depression polygenic risk scores improves the genetic prediction of lithium response in bipolar disorder patients**

**Supplementary Information**

**Authors**

Klaus Oliver Schubert, MD, PhD^1,2*^, Anbupalam Thalamuthu, PhD^3*^; Azmeraw T. Amare, MPH, MSc, PhD^1*^; Joseph Frank, PhD^4^; Fabian Streit, PhD^4^; The International Consortium of Lithium Genetics (ConLi^+^Gen); Mazda Adli, MD^5^; Nirmala Akula, PhD^6^; Kazufumi Akiyama, MD^7^; Raffaella Ardau, MD^8^; Bárbara Arias, PhD^9^; Jean-Michel Aubry, MD^10^; Lena Backlund, MD, PhD^11^; Abesh Kumar Bhattacharjee, MD^12^; Frank Bellivier, MD, PhD^13^; Antonio Benabarre, MD, PhD^14^; Susanne Bengesser, MD^15^; Joanna M. Biernacka, PhD^16^; Armin Birner, MD^15^; Cynthia Marie-Claire, PhD^13^; Micah Cearns, PhD^1^; Pablo Cervantes, MD^17^; Hsi-Chung Chen, MD, PhD^18^; Caterina Chillotti, MD^8^; Sven Cichon, PhD^19,20^; Scott R. Clark, MBBS, PhD ^1^; Cristiana Cruceanu, PhD^21^; Piotr M. Czerski, PhD^22^; Nina Dalkner, MSc^15^; Alexandre Dayer, MD^10^; Franziska Degenhardt, MD^20^; Maria Del Zompo, MD^23^; J. Raymond DePaulo, MD^24^; Bruno Étain, MD, PhD^13^; Peter Falkai, MD^25^; Andreas J. Forstner, MD^19,20,26^; Louise Frisen, MD^11^; Mark A. Frye, MD^16^; Janice M. Fullerton, PhD^27,28^; Sébastien Gard, MD^29^; Julie S. Garnham, BN^30^; Fernando S. Goes, MD^24^; Maria Grigoroiu-Serbanescu, PhD^31^; Paul Grof, MD, PhD^32^; Ryota Hashimoto, MD, PhD^33,34^; Joanna Hauser, MD^22^; Urs Heilbronner, PhD^35^; Stefan Herms, Dipl.Biol.^19,20^; Per Hoffmann, PhD^19,20^; Liping Hou, PhD^6^; Yi-Hsiang Hsu, PhD^36,37^; Stephane Jamain, PhD^38^; Esther Jiménez, PhD^14^; Jean-Pierre Kahn, MD, PhD^39^; Layla Kassem, PhD^6^; Po-Hsiu Kuo, PhD^40^; Tadafumi Kato, MD, PhD^41^; John Kelsoe, MD^12^; Sarah Kittel-Schneider, MD^42^; Ewa Ferensztajn-Rochowiak^43^; Barbara König, MSc^44^; Ichiro Kusumi, MD^45^; Gonzalo Laje, MD^6^; Mikael Landén, MD^46,47^; Catharina Lavebratt, PhD^11^; Marion Leboyer, MD, PhD^48^; Susan G. Leckband, BSc^49^; Mario Maj, MD^50^; Major Depressive Disorder Working Group of the Psychiatric Genomics Consortium^51^; Mirko Manchia, MD, PhD^52,53^; Lina Martinsson, MD^54^; Michael J. McCarthy, MD, PhD^12,55^; Susan McElroy, MD^56^; Francesc Colom, PhD^57,58,^; Marina Mitjans, PhD^59,60,61,^ ; Francis M. Mondimore, MD^24^; Palmiero Monteleone, MD^62,^; Caroline M. Nievergelt, PhD^12^; Markus M. Nöthen, MD^20^; Tomas Novák, MD^63^; Claire O’Donovan, MB^30^; Norio Ozaki, MD^64^; Urban Ösby, MD, PhD^65^; Sergi Papiol, PhD^35,66^; Andrea Pfennig, MD^67^; Claudia Pisanu, MD^23^; James B. Potash, MD, MPH^24^; Andreas Reif, MD^42^; Eva Reininghaus, MD^15^; Guy A. Rouleau, MD^68^; Janusz K. Rybakowski, MD^43^; Martin Schalling, MD^11^; Peter R. Schofield, PhD, DSc^27,28^; Barbara W. Schweizer, RN^24^; Giovanni Severino, MD^23^; Tatyana Shekhtman, MSc^12^; Paul D. Shilling, PhD^12^; Katzutaka Shimoda, MD^69^; Christian Simhandl, MD^70^; Claire M. Slaney, RN^30^; Alessio Squassina, PhD^23^; Thomas Stamm, MD^5^; Pavla Stopkova, MD^63^; Fasil Tekola-Ayele, PhD^71^; Alfonso Tortorella, MD^72^; Gustavo Turecki, MD^21^; Julia Veeh, PhD^42^; Eduard Vieta, MD, PhD^14^; Stephanie H. Witt, PhD^4^; Gloria Roberts, PhD^73^; Peter P. Zandi, PhD^74^; Martin Alda, MD^30^; Michael Bauer, MD,PhD^67^; Francis J. McMahon, MD^6^; Philip B. Mitchell, MD^73^; Thomas G. Schulze, MD^4,6,24,35,75^; Marcella Rietschel, MD^4^; Bernhard T. Baune, MD, PhD^76-78^

**Author affiliations:**

^1^Discipline of Psychiatry, School of Medicine, University of Adelaide, Adelaide, SA, Australia

^2^Northern Adelaide Local Health Network, Mental Health Services, Adelaide, SA, Australia

^3^Centre for Healthy Brain Ageing (CHeBA), School of Psychiatry, University of New South Wales, Sydney, Australia

^4^Department of Genetic Epidemiology in Psychiatry, Central Institute of Mental Health, Medical Faculty Mannheim, University of Heidelberg, Mannheim, Germany

^5^Department of Psychiatry and Psychotherapy, Charité - Universitätsmedizin Berlin, Campus Charité Mitte, Berlin, Germany

^6^Intramural Research Program, National Institute of Mental Health, National Institutes of Health, US Department of Health & Human Services, Bethesda, MD, USA

^7^Department of Biological Psychiatry and Neuroscience, Dokkyo Medical University School of Medicine, Mibu, Tochigi, Japan

^8^Unit of Clinical Pharmacology, Hospital University Agency of Cagliari, Cagliari, Italy

^9^Unitat de Zoologia i Antropologia Biològica (Dpt. Biologia Evolutiva, Ecologia i Ciències Ambientals), Facultat de Biologia and Institut de Biomedicina (IBUB), University of Barcelona, CIBERSAM, Barcelona, Spain

^10^Department of Psychiatry, Mood Disorders Unit, HUG - Geneva University Hospitals, Geneva, Switzerland

^11^Department of Molecular Medicine and Surgery, Karolinska Institute, Stockholm, Sweden, and Center for Molecular Medicine, Karolinska University Hospital, Stockholm, Sweden

^12^Department of Psychiatry, University of California San Diego, San Diego, CA, United States

^13^INSERM UMR-S 1144, Université Paris Diderot, Département de Psychiatrie et de Médecine Addictologique, AP-HP, Groupe Hospitalier Saint-Louis-Lariboisière-F.Widal, Paris, France

^14^Bipolar Disorder Program, Institute of Neuroscience, Hospital Clinic, University of Barcelona, IDIBAPS, CIBERSAM, Barcelona, Catalonia, Spain

^15^Department of Psychiatry and Psychotherapeutic Medicine, Research Unit for bipolar affective disorder, Medical University of Graz, Graz, Austria

^16^Department of Health Sciences Research, Mayo Clinic, Rochester, MN, United States

^16^Department of Psychiatry and Psychology, Mayo Clinic, Rochester, MN, United States

^17^The Neuromodulation Unit, McGill University Health Centre, Montreal, Canada

^18^Department of Psychiatry & Center of Sleep Disorders, National Taiwan University Hospital, Taipei, Taiwan

^19^Human Genomics Research Group, Department of Biomedicine, University Hospital Basel, Basel, Switzerland

^20^Institute of Human Genetics, University of Bonn and Department of Genomics, Life & Brain Center, Bonn, Germany

^21^Douglas Mental Health University Institute, McGill University, Montreal, Canada

^22^Psychiatric Genetic Unit, Poznan University of Medical Sciences, Poznan, Poland

^23^Department of Biomedical Sciences, University of Cagliari, Cagliari, Italy

^24^Department of Psychiatry and Behavioral Sciences, Johns Hopkins University, Baltimore, MD, United States

^25^Department of Psychiatry and Psychotherapy, Ludwig-Maximilian-University Munich, Munich, Germany

^26^Department of Psychiatry (UPK), University of Basel, Basel, Switzerland

^27^Neuroscience Research Australia, Sydney, NSW, Australia

^28^School of Medical Sciences, University of New South Wales, Sydney, NSW, Australia

^29^Service de psychiatrie, Hôpital Charles Perrens, Bordeaux, France

^30^Department of Psychiatry, Dalhousie University, Halifax, Nova Scotia, Canada

^31^Biometric Psychiatric Genetics Research Unit, Alexandru Obregia Clinical Psychiatric Hospital, Bucharest, Romania

^32^Mood Disorders Center of Ottawa, Ontario, Canada

^33^Molecular Research Center for Children's Mental Development, United Graduate School of Child Development, Osaka University, Osaka, Japan

^34^Department of Psychiatry, Osaka University Graduate School of Medicine, Osaka, Japan

^35^Institute of Psychiatric Phenomics and Genomics (IPPG), University Hospital, LMU Munich, Munich, Germany

^36^HSL Institute for Aging Research, Harvard Medical School, Boston, MA, United States

^37^Program for Quantitative Genomics, Harvard School of Public Health, Boston, MA, United States

^38^Univ Paris Est Créteil, INSERM, IMRB, Translational Neuropsychiatry, Fondation FondaMental, Créteil, France

^39^Service de Psychiatrie et Psychologie Clinique, Centre Psychothérapique de Nancy - Université de Lorraine, Nancy, France

^40^Department of Public Health & Institute of Epidemiology and Preventive Medicine,

College of Public Health, National Taiwan University, Taipei, Taiwan

^41^Department of Psychiatry & Behavioral Science, Juntendo University, Graduate School of Medicine, Tokyo, Japan

^42^Department of Psychiatry, Psychosomatic Medicine and Psychotherapy, University Hospital Frankfurt, Frankfurt, Germany

^43^Department of Adult Psychiatry, Poznan University of Medical Sciences, Poznan, Poland

^44^Department of Psychiatry and Psychotherapeutic Medicine, Landesklinikum Neunkirchen, Neunkirchen, Austria

^45^Department of Psychiatry, Hokkaido University Graduate School of Medicine, Sapporo, Japan

^46^Institute of Neuroscience and Physiology, the Sahlgrenska Academy at the Gothenburg University, Gothenburg, Sweden

^47^Department of Medical Epidemiology and Biostatistics, Karolinska Institutet, Stockholm, Sweden

^48^Inserm U955, Translational Neuro-Psychiatry laboratory, Université Paris Est Créteil (UPEC), AP-HP, Department of Psychiatry and Addictology of Mondor University Hospital, AP-HP, Fondation FondaMental, Créteil, France

^49^Office of Mental Health, VA San Diego Healthcare System, San Diego, CA, United States

^50^Department of Psychiatry, University of Campania “Luigi Vanvitelli”, Naples, Italy

^51^For a full list of major depressive disorder working group of the PGC investigators, see the Supplementary Material

^52^Section of Psychiatry, Department of Medical Sciences and Public Health, University of Cagliari, Cagliari, Italy

^53^Department of Pharmacology, Dalhousie University, Halifax, NS, Canada

^54^Department of Clinical Neurosciences, Karolinska Institutet, Stockholm, Sweden

^55^Department of Psychiatry, VA San Diego Healthcare System, San Diego, CA, United States

^56^Department of Psychiatry, Lindner Center of Hope / University of Cincinnati, Mason, OH, United States

^57^Mental Health Research Group, IMIM-Hospital del Mar, Barcelona, Catalonia, Spain

^58^Centro de Investigación Biomédica en Red de Salud Mental (CIBERSAM), Instituto de Salud Carlos III, Madrid, Spain

^59^Departament de Genètica, Microbiologia i Estadística, Facultat de Biologia, Universitat de Barcelona, Barcelona, Spain

^60^Institut de Biomedicina de la Universitat de Barcelona (IBUB), Barcelona, Spain

^61^Centro de Investigación Biomédica en Salud Mental (CIBERSAM), Madrid, Spain

^62^Department of Medicine, Surgery and Dentistry “Scuola Medica Salernitana”, University of Salerno, Salerno, Italy

^63^National Institute of Mental Health, Klecany, Czech Republic

^64^Department of Psychiatry & Department of Child and Adolescent Psychiatry, Nagoya University Graduate School of Medicine, Nagoya, Japan

^65^Department of Neurobiology, Care Sciences, and Society, Karolinska Institutet and Center for Molecular Medicine, Karolinska University Hospital, Stockholm, Sweden

^66^Department of Psychiatry and Psychotherapy, Ludwig-Maximilian-University Munich, Munich, Germany

^67^Department of Psychiatry and Psychotherapy, University Hospital Carl Gustav Carus, Medical Faculty, Technische Universität Dresden, Germany

^68^Montreal Neurological Institute and Hospital, McGill University, Montreal, Canada

^69^Department of Psychiatry, Dokkyo Medical University School of Medicine, Mibu, Tochigi, Japan

^70^Bipolar Center Wiener Neustadt, Sigmund Freud University, Medical Faculty, Vienna, Austria

^71^Epidemiology Branch, Division of Intramural Population Health Research, Eunice Kennedy Shriver National Institute of Child Health and Human Development, National Institutes of Health, Bethesda, MD, USA

^72^Department of Psychiatry, University of Perugia, Italy

^73^School of Psychiatry, University of New South Wales, Sydney, Australia

^74^Department of Mental Health, Johns Hopkins Bloomberg School of Public Health, Baltimore, MD, United States

^75^Department of Psychiatry and Psychotherapy, University Medical Center (UMG), Georg-August University Göttingen, Göttingen, Germany

^76^ Department of Psychiatry and Psychotherapy, University of Münster, Münster, Germany

^77^ Department of Psychiatry, Melbourne Medical School, University of Melbourne, Parkville, Victoria, Australia

^78^The Florey Institute of Neuroscience and Mental Health, The University of Melbourne

Parkville, VIC, Australia

*Klaus Oliver Schubert, Anbupalam Thalamuthu, and Azmeraw T. Amare contributed equally and should be regarded as joint first authors.

**Corresponding author**

Prof. Bernhard Baune, PhD, MD, MPH, MBA, FRANZCP

Head, Department of Mental Health

Director, Lab division of Molecular Neurobiology of Mental Health

University of Münster

Albert-Schweitzer Campus 1

Building A 9

48149 Münster

GERMANY

T +49251-83-56664

Email: [Bernhard.Baune@ukmuenster.de](mailto:Bernhard.Baune@ukmuenster.de)

**Contents**

[Supplementary figures and tables 8](#_Toc83480117)

[Supplementary figures 8](#_Toc83480118)

[Supplementary Figure 1: IPA® Top 2 Network of MET2 genes. 8](#_Toc83480119)

[Supplementary Figure 2: IPA® Top 3 Network (1 of 3) of MET2 genes. 9](#_Toc83480120)

[Supplementary Figure 3: IPA® Top 4 Network (2 of 3) of MET2 genes. 10](#_Toc83480121)

[Supplementary Figure 4: IPA® Top 5 Network (2 of 3) of MET2 genes. 11](#_Toc83480122)

[Supplementary Tables 12](#_Toc83480123)

[**Supplementary Table 1:** Summary statistics for the associations between PRS and response to lithium measured by a dichotomous outcome on the Alda scale, for bipolar disorder (BD), schizophrenia (SCZ), depression (MDD), meta-analysis of these 3 traits (MET3), and meta-analysis of SCZ and MDD (MET2). 12](#_Toc83480124)

[**Supplementary Table 2:** Summary statistics of the associations between PRScs and a continuous lithium treatment response measure (Alda Total), for bipolar disorder PRS (BD), schizophrenia PRS (SCZ), major depression PRS (MDD), meta-MDD/SCZ/BD PRS (MET3), and meta-MDD/SCZ PRS (MET2). 12](#_Toc83480125)

[**Supplementary Table 3:** Effect estimates and odd’s ratios (OR) for likelihood of poorer lithium response (Alda score ≤6). 12](#_Toc83480126)

[**Supplementary Table 4:** Pearson’s correlations between PRSs for bipolar disorder (BD), schizophrenia (SCZ), major depressive disorder (MDD), meta-BD/SCZ/MDD (MET3), and meta-SCZ/MDD(MET2). 13](#_Toc83480127)

[**Supplementary Table 5:** 8,619 SNPs obtained from the MDD & SCZ meta-analysis (MET2), with associations with SCZ and MDD at p<5x10^-8^ 13](#_Toc83480128)

[**Supplementary Table 6:** List of 270 hosting genes and nearby unique genes (MET2) 13](#_Toc83480129)

[**Supplementary Table 7**: Gene list and annotations of 256 MET2 genes used by IPA® for functional bioinformatic analysis of MET2 PRS. 13](#_Toc83480130)

[**Supplementary Table 8:** Summary of IPA® findings for functional- and pathway annotations of 256 MET2 genes. 13](#_Toc83480131)

[**Supplementary Table 9:** MET2 genes that were mapped to “insulin-dependent diabetes mellitus”, “diabetes mellitus” and “glucose metabolism disorder” pathways by IPA®. 14](#_Toc83480132)

# Supplementary figures and tables

## Supplementary figures

### Supplementary Figure 1: IPA® Top 2 Network of MET2 genes.


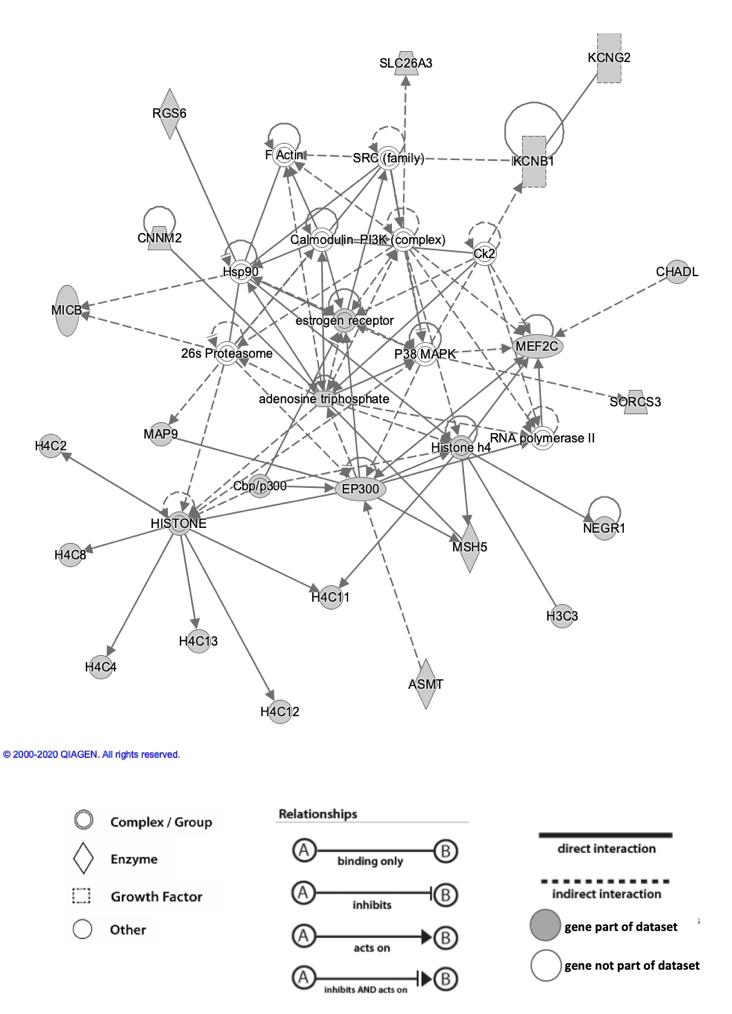


Legend Supplementary figure 1: **IPA® Top 2 Network of MET2 genes.** Annotated network functions include cell cycle, cellular assembly and organization, and DNA replication, recombination, and repair. Adenosine triphosphate (ATP), estrogen receptor, and EP300 (encoding p300, a histone acetyltransferase) are identified as nodes with most network interactions.

### Supplementary Figure 2: IPA® Top 3 Network (1 of 3) of MET2 genes.


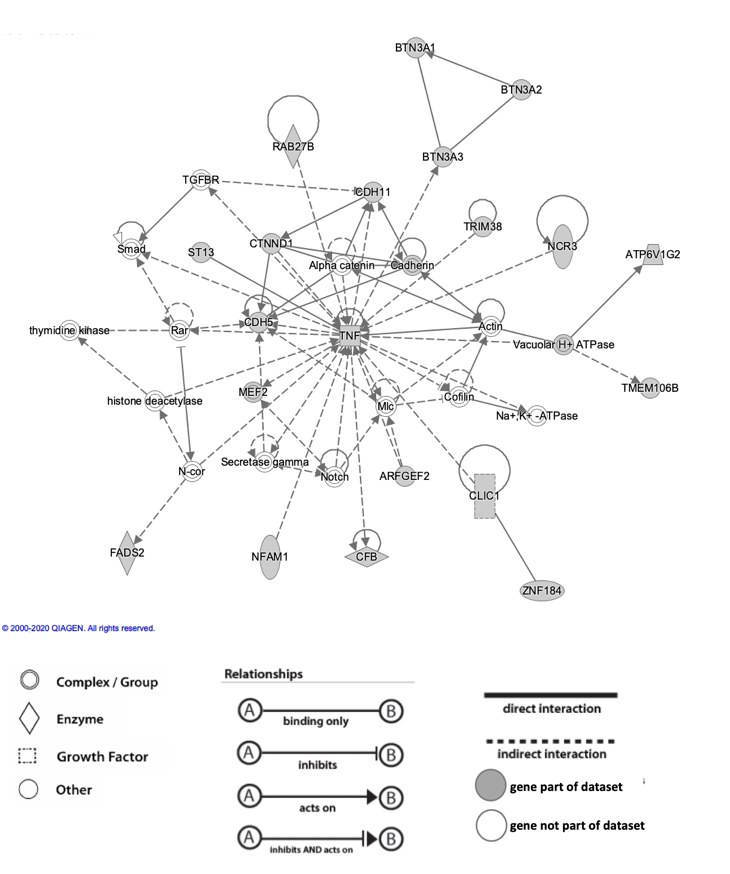


Legend Supplementary figure 2: **IPA® Top 3 Network (1 of 3) of MET2 genes.** Annotated network functions include cell death and survival, cell-to-cell signaling and interaction, and cellular assembly and organization. Tumor necrosis factor (TNF) is identified as node with most network interactions.

### Supplementary Figure 3: IPA® Top 4 Network (2 of 3) of MET2 genes.


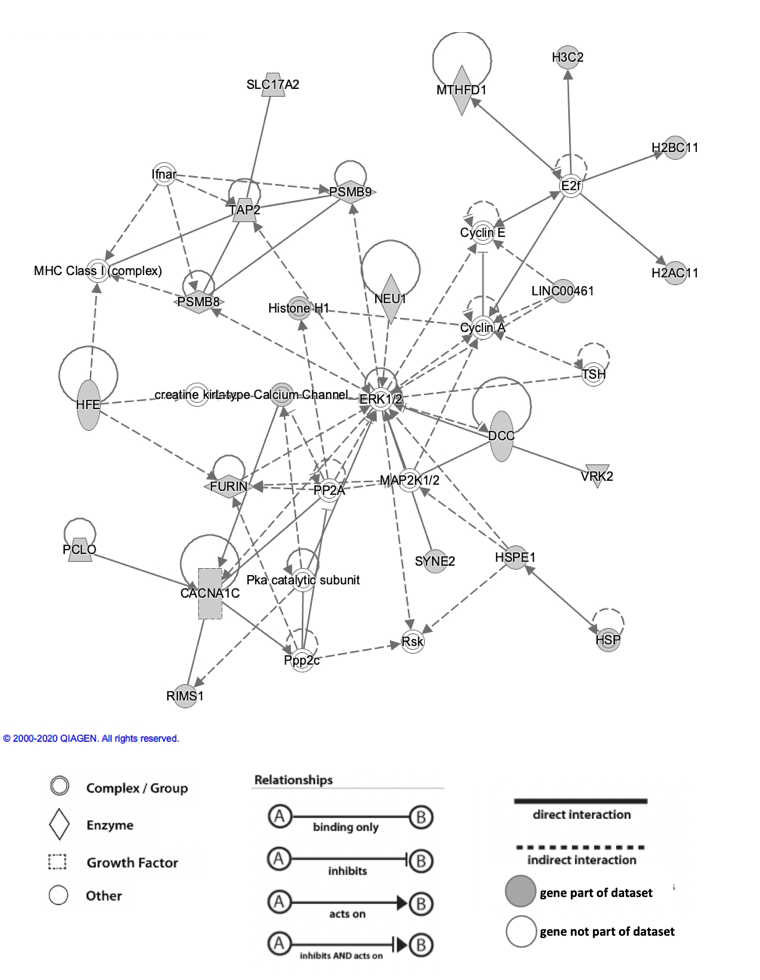


Legend Supplementary figure 3: **IPA® Top 4 Network (2 of 3) of MET2 genes.** Annotated network functions include infectious diseases, cancer, and hematological disease. Extracellular-signal-regulated kinases (ERK1/2) are identified as node with most network interactions.

### Supplementary Figure 4: IPA® Top 5 Network (2 of 3) of MET2 genes.


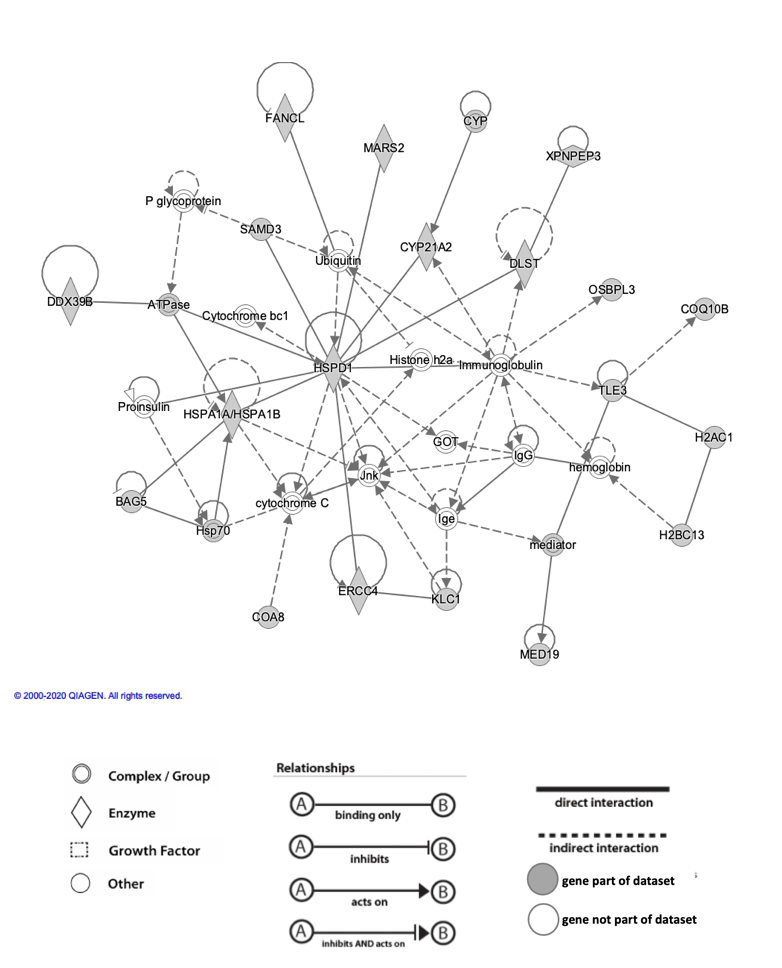


Legend Supplementary figure 4: **IPA® Top 5 Network (2 of 3) of MET2 genes.** Annotated network functions include Post-Translational Modification, Protein Folding, Protein Degradation. 60 kDa heat shock protein, mitochondrial (HSPD1) is identified as node with most network interactions.

## Supplementary Tables

### **Supplementary Table 1:** Summary statistics for the associations between PRS and response to lithium measured by a dichotomous outcome on the Alda scale, for bipolar disorder (BD), schizophrenia (SCZ), depression (MDD), meta-analysis of these 3 traits (MET3), and meta-analysis of SCZ and MDD (MET2).

**See excel file “Supplementary Tables”, tab 1**

Legend Supplementary Table 1: Responders were defined as those obtaining an Alda score of 7 or more. *GWAS:* genome-wide association data used for PRS generation; *OR*: odds ratio; *SE*: standard error; *tvalue*: t-value; *Pval*: p-value; *R2*: Nagelkerke R square. All analyses are adjusted for the co-variates age, gender, 4 principal components (PCs), site, and SNP chip type.

### **Supplementary Table 2:** Summary statistics of the associations between PRScs and a continuous lithium treatment response measure (Alda Total), for bipolar disorder PRS (BD), schizophrenia PRS (SCZ), major depression PRS (MDD), meta-MDD/SCZ/BD PRS (MET3), and meta-MDD/SCZ PRS (MET2).

**See excel file “Supplementary Tables”, tab 2**

Legend Supplementary Table 2: *GWAS*: genome-wide association data used for PRS generation; *SE*: standard error; *tvalue*: t-value; *Pval*: p-value; all analyses are adjusted for the co-variates age, gender, 4 principal components (PCs), site, and SNP chip type.

### **Supplementary Table 3:** Effect estimates and odd’s ratios (OR) for likelihood of poorer lithium response (Alda score ≤6).

**See excel file “Supplementary Tables”, tab 3**

Legend Supplementary Table 3: Patients were divided by decile groups, according to relative loading with the respective PRS. Decile group 1 (reference) has the lowest PRS loading, whereas Decile group 10 has the highest PRS loadings.

### **Supplementary Table 4:** Pearson’s correlations between PRSs for bipolar disorder (BD), schizophrenia (SCZ), major depressive disorder (MDD), meta-BD/SCZ/MDD (MET3), and meta-SCZ/MDD(MET2).

**See excel file “Supplementary Tables”, tab 4**

Legend Supplementary Table 4: see table heading

### **Supplementary Table 5:** 8,619 SNPs obtained from the MDD & SCZ meta-analysis (MET2), with associations with SCZ and MDD at p<5x10^-8^

**See excel file “Supplementary Tables”, tab 5**

Legend Supplementary Table 5: see table heading

### **Supplementary Table 6:** List of 270 hosting genes and nearby unique genes (MET2)

**See excel file “Supplementary Tables”, tab 6**

Legend Supplementary Table 6: List of 270 hosting genes and nearby unique genes (within 1MB) derived from treatment response-associated SNPs within MET2 (Supplementary Table 5), at meta-GWAS threshold p<5x10^-8^. Lists of genes intersecting between MET2, SCZ (at GWAS p-value ≤ 5x10^-8^), and MDD (at GWAS p-value ≤ 5x10^-8^).

### **Supplementary Table 7**: Gene list and annotations of 256 MET2 genes used by IPA® for functional bioinformatic analysis of MET2 PRS.

**See excel file “Supplementary Tables”, tab 7**

Legend Supplementary Table 7: see table heading

### **Supplementary Table 8:** Summary of IPA® findings for functional- and pathway annotations of 256 MET2 genes.

**See excel file “Supplementary Tables”, tab 8**

Legend Supplementary Table 8: see table heading

### **Supplementary Table 9:** MET2 genes that were mapped to “insulin-dependent diabetes mellitus”, “diabetes mellitus” and “glucose metabolism disorder” pathways by IPA®.

**See excel file “Supplementary Tables”, tab 9**

Legend Supplementary Table 9: see table heading
